# Improving search efficiency for systematic reviews of diagnostic test accuracy: an exploratory study to assess the viability of limiting to MEDLINE, EMBASE and reference checking

**DOI:** 10.1186/s13643-015-0074-7

**Published:** 2015-06-26

**Authors:** Louise Preston, Christopher Carroll, Paolo Gardois, Suzy Paisley, Eva Kaltenthaler

**Affiliations:** School of Health and Related Research (ScHARR), University of Sheffield, Sheffield, S1 4DA England; Department of Public Health and Pediatrics, University of Turin, Piazza Polonia, 94, 10125 Turin, Italy

**Keywords:** Systematic reviews, Diagnostic test accuracy, Literature searching, MEDLINE, EMBASE, Health Technology Assessment

## Abstract

**Background:**

Increasing numbers of systematic reviews evaluating the diagnostic test accuracy of technologies are being published. Currently, review teams tend to apply conventional systematic review standards to identify relevant studies for inclusion, for example sensitive searches of multiple bibliographic databases. There has been little evaluation of the efficiency of searching only one or two such databases for this type of review. The aim of this study was to assess the viability of an approach that restricted searches to MEDLINE, EMBASE and the reference lists of included studies.

**Methods:**

A convenience sample of nine Health Technology Assessment (HTA) systematic reviews of diagnostic test accuracy, with 302 included citations, was analysed to determine the number and proportion of included citations that were indexed in and retrieved from MEDLINE and EMBASE. An assessment was also made of the number and proportion of citations not retrieved from these databases but that could have been identified from the reference lists of included citations.

**Results:**

287/302 (95 %) of the included citations in the nine reviews were indexed across MEDLINE and EMBASE. The reviews’ searches of MEDLINE and EMBASE accounted for 85 % of the included citations (256/302). Of the forty-six (15 %) included citations not retrieved by the published searches, 24 (8 %) could be found in the reference lists of included citations. Only 22/302 (7 %) of the included citations were not found by the proposed, more efficient approach.

**Conclusions:**

The proposed approach would have accounted for 280/302 (93 %) of included citations in this sample of nine systematic reviews. This exploratory study suggests that there might be a case for restricting searches for systematic reviews of diagnostic test accuracy studies to MEDLINE, EMBASE and the reference lists of included citations. The conduct of such reviews might be rendered more efficient by using this approach.

## Background

Increasing numbers of systematic reviews evaluating diagnostic technologies are being published in the field of Health Technology Assessment (HTA). In response to the needs of policy-makers in this field, in the last years, the National Institute for Health and Care Excellence (NICE) has established a Diagnostics Assessment Programme and a Diagnostics Advisory Committee, having run a pilot project to develop methods in this area [1, 2]. Systematic reviews or individual studies of diagnostic test accuracy usually compare an index test with the best available test or current standard procedure for making a diagnosis. The methodological challenges of undertaking systematic reviews of diagnostic accuracy studies are well known and have been extensively discussed in the academic literature [3, 4]. Searching for and identifying evidence is one challenge when undertaking such a systematic review. Search filters, including validated filters, are available from various sources, but their use is now not recommended by some organisations because the results from applying these filters are variable [4, 5]. This is due in part to inconsistency in the reporting and indexing of papers. Consequently, diagnostic study filters compare less favourably with other search filters, e.g. for Randomised Controlled Trials [4]. The Cochrane Collaboration Diagnostic Test Accuracy Working Group is working on the publication of diagnostic test accuracy systematic reviews within the Cochrane Library and recognises the challenges of searching for diagnostic studies. The Cochrane Handbook for Systematic Reviews of Diagnostic Test Accuracy has a chapter on searching for studies, which recommends that “a range of databases be considered for searching”, including MEDLINE, EMBASE and regional databases (to account for the differing disease prevalence within different geographical regions) [6].

Within the information science community, there is a growing interest in search efficiency, in particular whether it is possible to identify the same sample of included studies for a systematic review by searching fewer databases than the traditionally large number deemed necessary [7, 8]. This perhaps inevitable move has been driven by several factors, including the improved indexing and searching capabilities of databases and the need to produce high-quality reviews within time and resource constraints [9]. Consequently, it has been argued that a well-structured search undertaken in only two or three databases (supported by additional methods to identify evidence, such as reference list checking, citation searching, contact with manufacturers and experts) might identify evidence more efficiently than a similar search undertaken in more databases [7].

Recent research evaluated whether searches for studies of diagnostic test accuracy for systematic review and meta-analysis could be limited to MEDLINE alone [10]. Appraising 44 reviews of diagnostic test accuracy studies containing 76 meta-analyses, the authors found that in 65 of the 76 meta-analyses (85.5 %), all of the studies were identifiable in MEDLINE. Of the remaining 11 meta-analyses, 87.5–99 % of the studies were identifiable in MEDLINE. Therefore, the authors suggest that extensive searching in databases other than MEDLINE has minimal effect on the identification of studies for inclusion in diagnostic reviews. However, this conclusion assumes that the actual searches undertaken in MEDLINE for all 44 reviews would have had 100 % sensitivity: that is, they would have retrieved all of the relevant studies indexed in that database. In a separate study by the same authors, statistical tests were also undertaken on a sub-set of those meta-analyses for which not all included studies were indexed in MEDLINE. This found that the omission of any of the “missed” studies would not have impacted on the basic findings of that sample, though precision might be slightly affected [11].

An earlier study [12] sought to estimate the yield of searches for studies of diagnostic test accuracy across seven different databases by re-running the searches as they were described for eight specific systematic reviews. Taking the included studies from these reviews, the authors created a gold standard set of included studies (*n* = 522) and then categorised them as follows: 1) being indexed in the databases and retrieved by the published searches as they were described; 2) being indexed in the databases but not retrieved by those searches; and 3) not being indexed in any of the databases. The study found that no search identified all of the included studies in the gold standard set for any one of the eight reviews—even across all seven databases; that more than 20 % of the studies in any review were not identified by the search of MEDLINE (EMBASE, Science Citation Index and BIOSIS all contained studies that were not in MEDLINE); that another 22/522 were not retrieved from any of the seven databases using the reported searches, and that 8/522 studies were not indexed in any of the seven databases.

Given the different findings of these two studies [10, 12] (i.e. the potential value of MEDLINE alone vs the requirement to search multiple databases), there is a strong case for further exploratory research in the area of searching for diagnostic test accuracy studies for systematic reviews.

The aim of this study is therefore to examine whether it would be worthwhile to limit searching for diagnostic test accuracy studies to MEDLINE and EMBASE alone (rather than searching a longer list of databases), along with the standard systematic review supplementary technique of checking the references of included citations and relevant reviews. This is the proposed strategy. MEDLINE and EMBASE have been chosen as they are the two major general bibliographic databases in the health sciences and have been found to be the most important sources of evidence in Health Technology Assessment [13]. They are routinely recommended as a minimum for searches by bodies such as the Cochrane Collaboration [6] and NICE [1], and they are the databases with the majority of published search filters. The addition of reference checking, as a supplementary method, is also being assessed because it should be a standard technique to identify literature in all systematic reviews but its value as a search strategy has not yet been evaluated by previous research into systematic reviews of diagnostic test accuracy.

The specific objectives of this study therefore are to analyse a convenience sample of systematic reviews of diagnostic test accuracy studies in order to: 1) identify which citations were indexed on MEDLINE or EMBASE; 2) to identify the number and proportion of citations that were retrieved by the MEDLINE and EMBASE search strategies reported for these reviews; 3) to identify the number and proportion of studies that could have been retrieved by the searches of MEDLINE and EMBASE plus reference checking of studies identified as relevant (any that could not be found by this proposed strategy are referred to as “missing” citations); and, finally, 4) to detail the reported search strategies and consider implications for literature searching for systematic reviews of diagnostic test accuracy.

## Methods

### Identification of reviews and included citations indexed on MEDLINE or EMBASE

We used a convenience sample test-set of nine Health Technology Assessment systematic reviews of diagnostic test accuracy undertaken at one centre: the School for Health and Related Research (ScHARR) at the University of Sheffield, UK. We selected these reviews because the authors work at the same centre and were therefore able to access full details of the searches. This represented all of the diagnostic reviews undertaken for the National Institute for Health Research (NIHR) HTA and NICE programmes by this centre. These reviews were published between 2004 and 2014 and covered topics ranging from neonatal screening to diagnostic tools for breast cancer, heart and liver disease and stroke [5, 14–21]. For each systematic review, we identified the included citations and searched for them to ascertain whether they were indexed in MEDLINE and/or EMBASE regardless of whether they had been retrieved by the reported searches.

### Identification of included citations retrieved from MEDLINE and EMBASE by the reported search strategies

For each systematic review, we also identified the original MEDLINE and EMBASE search strategies either from the reports or from the in-house project folders. Where multiple search strategies were available, we chose the search strategy for the systematic review of diagnostic test accuracy (as opposed to modelling, prevalence etc.). We re-ran searches in June 2013 in MEDLINE (Ovid platform) and EMBASE using the original search strategies and date limits. The results were imported into EndNote X1. This permitted an assessment of the proportion of the citations in each review that were identified by the published searches of MEDLINE and EMBASE. The reports’ original Reference Manager libraries were checked to identify the source of any included studies that were not retrieved by these searches of MEDLINE or EMBASE.

### Identification of included citations not retrieved from MEDLINE or EMBASE

The reference lists of included citations retrieved by the searches reported for the respective reviews were also checked by one author (CC) to determine the proportion of non-retrieved citations that could still have been identified using this standard, systematic review searching method. Any citations that could not be found by the proposed strategy of searching MEDLINE, EMBASE and reference lists are listed as “missing studies”.

### The search strategies

**Fig. 1:**
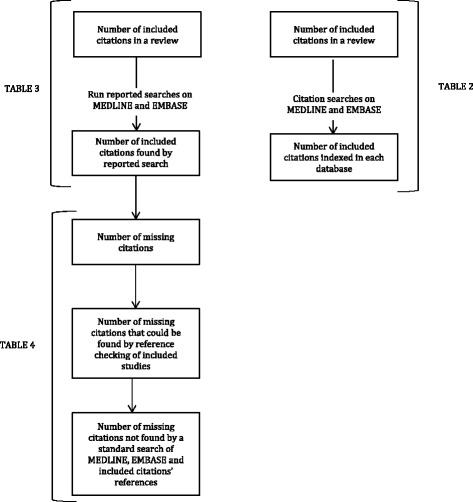
Summary of stages undertaken for each diagnostic review

Finally, we detailed the basic elements of the search strategies in terms of population and diagnostic test, plus any limitations such as filters, language and date. This enabled us to suggest reasons why included studies might have been missed by the reported searches of MEDLINE or EMBASE. Figure 1 describes the process.

## Results

### Characteristics of included reviews

We examined nine systematic reviews, published between 2004 and 2014. The total number of included citations was 302. The mean number of included studies in these reviews was 34 (range 15–51). A total number of 11 different databases were searched for evidence for the reviews. In terms of the number of databases searched per review, one review searched ten databases, five reviews searched a total of nine databases, two reviews searched eight databases and one review searched seven databases. All reviews searched a minimum of the following seven databases: MEDLINE, EMBASE, Central Register of Controlled Trials (CENTRAL), Cochrane Database of Systematic Reviews, NHS Database of Abstracts of Reviews of Effects (DARE), Health Technology Assessment (HTA) database and Web of Science (including the Science Citation Index and Conference Proceedings Citation Index). Cumulative Index of Nursing and Allied Health Literature was also searched for seven reviews, BIOSIS Previews for six reviews and PsycINFO and Health Management Information Consortium for one review each. See Table 1.

**Table 1:** Included reviews

Review	Topic	Number of included studies	Number and names of databases searched
Holmes (2014)	Routine echocardiography in the management of stroke and transient ischemic attack (TIA)	51	(*n* = 9) MEDLINE^a^, EMBASE, CENTRAL, CDSR, NHS DARE, HTA, CINAHL, Web of Science, PsycINFO
Ward (2013)	Gene expression profiling and expanded immunohistochemistry tests to guide the use of adjuvant chemotherapy in breast cancer	32	(*n* = 8) MEDLINE^a^, EMBASE, CENTRAL, CDSR, NHS DARE, HTA, BIOSIS, Web of Science
Simpson (2013)	Echocardiography in newly diagnosed atrial fibrillation patients	44	(*n* = 7) MEDLINE^a^, EMBASE, CENTRAL, CDSR, NHS DARE, HTA, Web of Science
Goodacre (2013)	Diagnostic strategies for suspected acute coronary syndrome	40	(*n* = 8) MEDLINE^a^, EMBASE, CENTRAL, CDSR, NHS DARE, HTA, CINAHL, Web of Science
Stevenson (2012)	Non-invasive diagnostic assessment tools for the detection of liver fibrosis in patients with suspected alcohol-related liver disease	17	(*n* = 9) MEDLINE^a^, EMBASE, CENTRAL, CDSR, NHS DARE, HTA, CINAHL, Web of Science, BIOSIS
Cooper (2011)	Imaging for the assessment of axillary lymph node metastases in early breast cancer	45	(*n* = 9) MEDLINE^a^, EMBASE, CENTRAL, CDSR, NHS DARE, HTA, CINAHL, Web of Science, BIOSIS
Sutcliffe (2009)	Classical and novel biomarkers as prognostic risk factors for localised prostate cancer	30	(*n* = 9) MEDLINE^a^, EMBASE, CENTRAL, CDSR, NHS DARE, HTA, CINAHL, Web of Science, BIOSIS
Pandor (2004)	Neonatal screening for inborn errors of metabolism using tandem mass spectrometry	15	(*n* = 10) MEDLINE^a^, EMBASE, CENTRAL, CDSR, NHS DARE, HTA, CINAHL, Web of Science, BIOSIS, HMIC
Kaltenthaler (2004)	magnetic resonance cholangiopancreatography compared with diagnostic endoscopic retrograde cholangiopancreatography	28	(*n* = 9) MEDLINE^a^, EMBASE, CENTRAL, CDSR, NHS DARE, HTA, CINAHL, Web of Science, BIOSIS
	Total	302	

^a^Including MEDLINE in-process and other non-indexed citations

*CENTRAL* Cochrane Central Database of Controlled Trials, *CDSR* Cochrane Database of Systematic Reviews, *DARE* NHS Database of Abstracts of Reviews of Effects, *HTA* Health Technology Assessment database, *CINAHL* Cumulative Index of Nursing and Allied Health Literature, *various databases* Web of Science, *BIOSIS* previews/biological abstracts, *HMIC* Health Management Information Consortium

### Number and proportion of included citations indexed on MEDLINE or EMBASE

The nine reviews included 302 unique citations. Of these, 275 (91 %) were indexed in MEDLINE and 277 (92 %) were indexed in EMBASE (see Table 2). In any given review, the percentage of studies included in the review that were indexed in MEDLINE ranged from 72 to 100 % and in EMBASE ranged from 66 to 100 %. Across both databases, it ranged from 85 to 100 %. Across the 302 citations, 287 (95 %) were indexed in either MEDLINE or EMBASE or both. Of the 287 indexed studies, 265 (88 %) studies were indexed in both databases and so could have been found by searching just one of them: ten studies were unique to MEDLINE and 12 were unique to EMBASE. In five of the nine reviews, all of the included citations were indexed in either MEDLINE or EMBASE; in one review, only two citations were not indexed in either database; in two reviews, only four were not indexed; and in a single review, five were not indexed.

**Table 2:** Included citations indexed in MEDLINE and EMBASE (*n*/%)

Project	Included studies (*n* = )	Included studies indexed in MEDLINE	Included studies indexed in EMBASE	Included studies indexed in both MEDLINE and EMBASE	Included studies indexed in one database but not the other: MEDLINE/EMBASE	Included studies not indexed in either MEDLINE or EMBASE
Holmes (2014)	51	48 (94 %)	51 (100 %)	48/51 (94 %)	0/3	0
Ward (2013)	32	23 (72 %)	27 (84 %)	22/32 (69 %)	1/5	4
Simpson (2013)	44	44 (100 %)	44 (100 %)	44/44 (100 %)	0/0	0
Goodacre (2013)	40	38^a^ (95 %)	40 (100 %)	38/40 (95 %)	0/2	0
Stevenson (2012)	17	12 (80 %)	13 (87 %)	12/17 (80 %)	0/1	4
Cooper (2011)	45	40 (89 %)	39 (87 %)	39/45 (87 %)	1/0	5
Sutcliffe (2009)	30	29 (97 %)	30 (100 %)	29/30 (97 %)	0/1	0
Pandor (2004)	15	13 (87 %)	10 (66 %)	10/15 (66 %)	3/0	2
Kaltenthaler (2004)	28	28 (100 %)	23 (82 %)	23/28 (82 %)	5/0	0
Total	302	275 (91 %)	277 (92 %)	265 (88 %)	10/12	15 (5 %)

^a^One had not been fully published at the time of the report but existed as an “epub”, but it would have been retrieved by the strategy

### Number and proportion of included citations retrieved by the reported MEDLINE and EMBASE search strategies

The number of citations identified in MEDLINE or EMBASE using the reported search strategies for each review was lower than the number of indexed citations that could have been potentially identified (i.e. those indexed in MEDLINE or EMBASE but not retrieved by the reported searches). Across all reviews, the percentage of included citations retrieved by the published search strategies ranged from 60 to 100 % in MEDLINE and from 18 to 87 % in EMBASE. The proportion of citations found by the searches across both MEDLINE and EMBASE in the reviews ranged from 60 to 100 %. In total, the searches of MEDLINE and EMBASE identified 256/302 (85 %) of the included citations in these nine systematic reviews (see Table 3). The searches undertaken in MEDLINE and EMBASE found 100 % of the included citations in one review (Kaltenthaler [21]) and only missed a single citation in two reviews (Simpson [15], Sutcliffe [19]); in five reviews, between five and seven citations were not found by the searches. Only the MEDLINE or EMBASE searches undertaken for one review, Holmes [5], missed a sizeable number (14/51 = 27 %) of its included citations (even though all 14 citations were actually indexed in those databases, see Table 2).

**Table 3:** Included studies found by reported searches of MEDLINE and EMBASE (*n*/%)

Project	Included studies	Included studies identified in MEDLINE via search	Included studies identified in EMBASE via search	Total included studies retrieved by search across both MEDLINE and EMBASE	Total included studies indexed in MEDLINE and EMBASE but missed by the reported searches
Holmes (2014)	51	31 (61 %)	25 (49 %)	37/51 (73 %)	14/51
Ward (2013)	32	20 (63 %)	25 (78 %)	26/32 (81 %)	2/32
Simpson (2013)	44	43 (98 %)	24 (55 %)	43/44 (98 %)	1/44
Goodacre (2013)	40	27 (68 %)	27 (68 %)	34/40 (85 %)	6/40
Stevenson (2012)	17	11 (65 %)	11 (65 %)	12/17 (71 %)	1/17
Cooper (2011)	45	38 (84 %)	34 (76 %)	38/45 (84 %)	2/45
Sutcliffe (2009)	30	27 (90 %)	26 (87 %)	29/30 (97 %)	1/30
Pandor (2004)	15	9 (60 %)	4 (27 %)	9/15 (60 %)	4/15
Kaltenthaler (2004)	28	28 (100 %)	5 (18 %)	28/28 (100 %)	0/28
Total	302	234 (77 %)	181 (60 %)	256 (85 %)	31 (11 %)

### Sources of citations not retrieved from MEDLINE and EMBASE using the reported searches

The reported searches of MEDLINE and EMBASE therefore failed to identify 46 out of the 302 total included citations (15 %) (see Table 3). Thirty-one of these citations (11 %) were indexed in either MEDLINE or EMBASE but were not retrieved by these searches. The number of citations not retrieved by the MEDLINE or EMBASE searches varied by review from 0 to 14. For all but one of the reviews, additional sources were used to locate the included citations in each review (Table 4). The most common reported sources of citations not identified via MEDLINE or EMBASE was searching of reference lists (16 papers) and the Web of Science database (Science Citation Index) (14 papers). Other databases that were a source of included papers were BIOSIS (3 papers), PubMed (1 paper) and CINAHL (1 paper). The four databases available via the Cochrane Library (CENTRAL, CDSR, DARE and HTA), plus PsycINFO and Health Management Information Consortium did not identify any unique citations for these nine systematic reviews of diagnostic test accuracy studies.

**Table 4:** Reported and potential sources of citations not retrieved from MEDLINE and EMBASE (*n*/%)

Project	Included studies not retrieved by search of both MEDLINE/EMBASE	Sources of non-retrieved citations as reported in the reviews	Non-retrieved citations identifiable from reference lists of retrieved studies and reviews	Total identifiable from reported searches of MEDLINE, EMBASE, plus reference checking of included studies and reviews	Remaining citations published as abstracts only	“Missing” citations^a^
Holmes (2014)	14/51	Reference lists (11), WoS (2), Google (1)	13	50/51 (98 %)	0	1
Ward (2013)	6/32	Manufacturer (1), NR (5)	2	28/32 (88 %)	2	2
Simpson (2013)	1/44	NR (1)	0	43/44 (98 %)	0	1
Goodacre (2013)	6/40	WoS (4), CINAHL (1), personal contact (1)	5	39/40 (98 %)	0	1
Stevenson (2012)	5/17	WoS (3), Manufacturer (1), BIOSIS previews (1)	1	13/17 (76 %)	3	1
Cooper (2011)	7/45	WoS (3), BIOSIS previews (2), reference lists (1), PubMed (1)	0	38/45 (84 %)	5	2
Sutcliffe (2009)	1/30	WoS (1)	0	29/30 (97 %)	0	1
Pandor (2004)	6/15	Reference lists (4), WoS (1), NR (1)	3	12/15 (80 %)	2	1
Kaltenthaler (2004)	0/28	None	0	28/28 (100 %)	0	0
Total	46/302 (15 %)	Reference lists (16), WoS (14), NR (7), BIOSIS (3), others (6)	24/46 (52 %)	280/302 (93 %)	12/46 (26 %)	10/46 (22 %)

*WoS* Web of Science, *NR* not reported

^a^Citations not retrieved by the reported searches of MEDLINE or EMBASE or included after reference checking of any of these retrieved citations or relevant reviews

### “Missing” citations

For the purposes of this study, an assessment was also made to determine the proportion of non-retrieved citations that could have been identified from the references of retrieved citations: an approach which should be part of any systematic review search strategy (Table 4). This assessment found that 24/46 (52 %) of these citations were in the reference lists of other included citations in their respective reviews and so should or could have been found by this method. The proposed strategy of MEDLINE, EMBASE and reference checking therefore could have identified 280 (93 %) of the 302 citations included in the review sample. Consequently, there were 22/302 (7 %) “missing citations”. Of these, 12/46 (26 %) were abstracts not indexed in MEDLINE or EMBASE so could not be retrieved by searches of these databases. The other ten citations (3 % of the total citations across all reviews) were eight standard journal articles, a book chapter and an unpublished study submitted by the manufacturer [14].

### Details of the reported MEDLINE and EMBASE search strategies

The MEDLINE and EMBASE search strategies were recorded in the reports. The strategies were all constructed following standard techniques, breaking-down the search into its constituent parts and combining them in an appropriate manner: population; diagnostic test (index test); and, in some of these cases, a published filter for diagnostic studies or other restrictions [6]. In every review, for each part of the strategy, both free-text and, where appropriate, MeSH terms were used. See Table 5. None of the searches included terms for comparator tests (best available or current standard procedure) or outcomes. As a result, they were all arguably relatively sensitive searches. Some reviews applied few restrictions (and therefore achieved greater potential sensitivity) if the numbers retrieved were relatively small (e.g. [17, 20, 21]). The final column of Table 5 also demonstrates that MEDLINE and EMBASE could be responsible for as little 40 % [14] or as much as 100 % [21] of the total retrieved citations that needed screening. These databases accounted for 76 % of the citations screened across the nine reviews. A sizeable number of citations (24 %) might therefore have been removed from the study screening process by reducing the number of databases: across the nine reviews, after de-duplication of records in the reference management databases, 13,883 citations were screened that were not retrieved from either MEDLINE or EMBASE and could only have generated, as a maximum, 18 out of the 22 “missing” citations (two were provided by a manufacturer, one was identifiable by personal communication only and one from Google).

**Table 5:** Basic details of MEDLINE search strategies

Report	Population		Test		Filter	Other, e.g. date	Totals retrieved (*n* = MEDLINE and EMBASE^a^/all databases^b^)
	MesH	Free-text	MeSH	Free-text			
Holmes (2014)	Yes	Yes	Yes	Yes	Yes	No	12,006/13,075 (92 %)
Ward (2013)	Yes	Yes	Yes	Yes	No	2009 onwards	2415^a^/5990 (40 %)
Simpson (2013)	Yes	Yes	Yes	Yes	Yes	Human only	12,816/15,824 (81 %)
Goodacre (2013)	Yes	Yes	Yes	Yes	Yes	Human only, 1995 onwards	1607/2865 (56 %)
Stevenson (2012)	No	Yes	Yes	Yes	Yes	No	2265^a^/4039 (56 %)
Cooper (2011)	Yes	Yes	Yes	Yes	Yes	No	377/646 (58 %)
Sutcliffe (2009)	Yes	Yes	No	No	Yes	No	10,070^a^/12,963 (78 %)
Pandor (2004)	Yes	Yes	Yes	Yes	No	No	108^a^/145 (74 %)
Kaltenthaler (2004)	No	No	Yes	Yes	No	No	1437^a^/1437 (100 %)
							43,101/56,984 (76 %)

^a^Either from the re-run of searches in June 2013 or the project reference management databases, both with duplicates removed

^b^This number is taken from the final review’s PRISMA flow diagram and is usually with duplicates removed

## Discussion

The sample of systematic reviews covered here searched between seven and nine databases although some of these databases are principally or exclusively index systematic reviews (CDSR, DARE and HTA) and so were unlikely to produce many individual diagnostic test accuracy studies. However, the reported searches of MEDLINE and EMBASE alone, plus the checking of the reference lists of relevant papers, would have accounted for 280 (93 %) of the total included citations across all nine reports and 100 % of the included citations in four of the nine reports ([15, 16, 19, 21]).

In terms of indexed citations, the findings for MEDLINE (91 %) are similar to those reported by Van Enst and colleagues [10]. However, this percentage does not indicate what was identified by the searches that were developed and run for these particular reviews but only what could potentially have been identified based on the proportion of indexed citations. In the present study, the proportion of citations found by the actual searches across both MEDLINE and EMBASE in the reviews ranged from 60 to 100 %. Consequently, the evidence of this sample suggests that, on the whole, MEDLINE alone cannot be relied on to act as a single source database for systematic reviews of studies of diagnostic test accuracy.

The reported searches were constructed according to standard principles, but more sensitive searches might have had the potential to identify all of the indexed and included citations in each of these four reviews and to miss only the 15 non-indexed citations across the other five reviews. Searches could have been made more sensitive by the addition of further keywords or free-text terms or the removal of certain terms or sets of terms: for example, the reports by Kaltenthaler [21] and Sutcliffe [19] did not use filters, and the former did not use terms for the population and the latter did not use terms for the index test (Table 5). However, a more sensitive search would have also increased the number of hits and the size of the task involved in screening citations for inclusion, which can create practical problems for Health Technology Assessments which are required to produce reports within time constraints [9, 22]. For example, the searches conducted in MEDLINE and EMBASE for the review by Holmes et al. [5] only retrieved 37 of a possible 51 of the included citations (73 %) that were indexed in these databases. This represents a relatively low retrieval rate. The search strategy does appear to have been less sensitive than most other reviews with the exception of those by Simpson [15] and Goodacre [16], which applied the same or more limitations in terms of including all possible elements of a search (see Table 5). However, the searches from this review also generated the second largest number of citations for screening (13,075); the largest was one of the reviews with the least sensitive searches: Simpson [15] with 15,824 citations. The need to maintain manageable numbers for study selection screening would explain the development of these less sensitive searches.

Although the use of filters is not recommended for reviews of diagnostic test accuracy studies, some of the reviews in this sample pre-date this guidance from Cochrane and NICE. More importantly, it should be noted that the aims of Health Technology Assessments differ from Cochrane reviews: HTA reports address questions that are more complex than just a question of diagnostic accuracy, for example, the opportunity costs of implementing diagnostic strategies vs going straight to treatment. It is often strategies that are being compared, not just tests. So, in many of these reports, there are a number of other questions also being addressed and searched for, including adverse events, quality of life and cost-effectiveness. Working within time and resource constraints to produce such reports might require a more pragmatic approach to searching, such as the application of filters, when otherwise sensitive searches produce unmanageable numbers of citations [8, 22].

Twenty-two citations (7 %) could not be identified by the proposed method of searching just MEDLINE, EMBASE and the references of retrieved citations. Twelve of these citations were abstracts. Published abstracts should not be ignored, especially because studies included in systematic reviews of diagnostic test accuracy can take the form of ad hoc analyses rather than registered trials. They might offer key data for assessing and managing publication bias [23] and, for some topics, these data might be vital for a review’s findings, especially for tests about which little has been published, for instance if the technique is novel [24, 25].

It is true that the usefulness of abstracts might be limited by their lack of detail, which can prevent a meaningful assessment of risk of bias and can render data more uncertain. However, in this case study, all of the abstracts missed by the searches of MEDLINE, EMBASE and the reference lists did satisfy the reviews’ inclusion criteria and were used in their analyses. It should be mentioned also that the majority of the reviews performed narrative synthesis rather than meta-analysis, so the impact of their possible omission is difficult to quantify. However, given the very small proportion of “missing” studies, their impact on the findings on the respective reviews is likely to have been minimal.

The diagnostic topics covered by the nine systematic reviews were diverse and were undertaken over an extended period (2004–2014), so, other than their conduct by a single centre, this does not represent a particularly restricted sample. This evidence indicates that an approach that involves searching MEDLINE and EMBASE using strategies constructed by applying standard systematic review techniques, then carefully checking the references of included papers, is likely to be more than sufficient for a systematic review of diagnostic studies. In this way, only 22/302 (7 %) of citations would have been missed across nine reviews and, in four reviews, no citations would be missed at all. Such a level of omission is unlikely to adversely affect the findings of systematic reviews of diagnostic test accuracy: it has been demonstrated that a larger percentage of missed studies had little effect on meta-analyses of a sample of diagnostic test accuracy reviews [11]. This approach would also save a great deal of time and effort and, given the smaller numbers of citations needing screening, would possibly also reduce the risk of reviewer error in selecting citations for potential inclusion. This would permit a more rapid evidence synthesis, whilst not compromising systematic review principles or increasing the risk of bias [22].

### Limitations

This study used a small, non-random sample of diagnostic test accuracy systematic reviews. This was done for reasons of pragmatism: first, because the authors had full access to the search strategies and reference databases of these reviews and, second, because of the exploratory nature of this project. We also assumed that the vast majority of the included citations in the reviews were located through screening of titles, abstracts and full papers. We have also assumed, because the number of studies missed by operating the proposed MEDLINE, EMBASE and reference tracking strategy is so small that the findings of the systematic reviews would not have been greatly affected by their omission. However, this is uncertain and can only be assessed statistically by excluding those particular studies from the many analyses reported in the reviews, although, as noted above, most of these reviews conducted narrative synthesis. Such an analysis is a major task to undertake retrospectively and has therefore not been completed in this exploratory study. Future work should test the findings of this small study in a larger, preferably prospective sample of systematic reviews from multiple institutions. If possible, statistical analysis should also be undertaken to quantify fully the impact of omitting any data from studies that might otherwise be missed [11].

## Conclusions

This small study seeks to add to the otherwise limited amount of research in this field. It differs from Van Enst and colleagues [10] by indicating that systematic reviews of diagnostic test accuracy studies do require searching of both MEDLINE and EMBASE, rather than MEDLINE alone, if they are to successfully identify all or almost all relevant citations. It also differs from Whiting and colleagues [12] by indicating that a search conducted using MEDLINE and EMBASE alone, supplemented by standard reference checking, was able to successfully identify all or almost all relevant citations: The searching of multiple databases is therefore not required. Depending on the topic, the search of a database of conference proceedings might also be required. The process therefore becomes more simple, contained and arguably more efficient, whilst not increasing the risk of bias or compromising the key principles of systematic review.

## Abbreviations

CDSR: Cochrane Database of Systematic Reviews
CENTRAL: Central Register of Controlled Clinical Trials
DARE: Database of Abstracts of Reviews of Effects
HTA: Health Technology Assessment
NICE: National Institute for Health and Care Excellence
NIHR: National Institute for Health Research
RCT: Randomised Controlled Trial

## References

[1] National Institute for Health and Clinical Excellence Centre for Health Technology Evaluation Diagnostics Assessment Programme (2011). Report on pilot project on the assessment of non-invasive diagnostic assessment tools for the detection of liver fibrosis in patients with suspected alcohol related liver disease.

[2] National Institute for Health and Clinical Excellence (2011). Diagnostics Assessment Programme manual.

[3] Whiting P, Rutjes AW, Westwood M, Mallett S, Deeks JJ, Reitsma JB (2011). QUADAS-2: a revised tool for the quality assessment of diagnostic accuracy studies. Ann Intern Med.

[4] Leeflang M, Deeks JJ, Gatsonis C, Bossuyt P (2008). Cochrane Diagnostic Test Accuracy Working Group. Systematic reviews of diagnostic test accuracy. Ann Intern Med.

[5] Holmes M, Rathbone J, Littlewood C, Rawdin A, Stevenson M, Stevens JW (2014). Routine echocardiography in the management of stroke and transient ischemic attack (TIA): a systematic review and an economic evaluation. Health Technol Assess.

[6] de Vet HCW, Eisinga A, Riphagen II, Aertgeerts B, Pewsner D (2008). Chapter 7: Searching for Studies. Cochrane Handbook for Systematic Reviews of Diagnostic Test Accuracy Version 0.4 [updated September 2008]. The Cochrane Collaboration.

[7] Beyer F, Wright K (2012). Can we prioritise which databases to search? A case study using a systematic review of frozen shoulder management. Health Info Lib J.

[8] Booth A (2010). How much searching is enough? Comprehensive versus optimal retrieval for technology assessments. Int J Technol Assess Health Care.

[9] Egger M, Juni P, Bartlett C, Holenstein F, Sterne J (2003). How important are comprehensive literature searches and the assessment of trial quality in systematic reviews? Empirical study. Health Technol Assess.

[10] Van Enst WA, Scholten RJPM, Hooft L (2011). Could a search for a diagnostic test accuracy review be restricted to MEDLINE?.

[11] Van Enst WA, Scholten RJPM, Whiting P, Zwinderman A, Hooft L (2014). Meta-epidemiologic analysis indicates that MEDLINE searches are sufficient for diagnostic test accuracy systematic reviews. J Clin Epidem.

[12] Whiting P, Westwood M, Burke M, Sterne J, Glanville J (2008). Systematic reviews of test accuracy should search a range of databases to identify primary studies. J Clin Epidem.

[13] Royle P, Waugh N (2003). Literature searching for clinical and cost-effectiveness studies used in health technology assessment reports carried out for the National Institute for Clinical Excellence appraisal system. Health Technol Assess.

[14] Ward SE, Scope A, Rafia R, Pandor A, Harnan S, Evans P (2013). Gene expression profiling and expanded immunohistochemistry tests to guide the use of adjuvant chemotherapy in breast cancer management: a systematic review and cost-effectiveness analyses. Health Technol Assess.

[15] Simpson E, Stevenson M, Scope A, Poku E, Minton J, Evans P (2013). Echocardiography in newly diagnosed atrial fibrillation patients: a systematic review and economic evaluation. Health Technol Assess.

[16] Goodacre S, Thokala P, Carroll C, Stevens JW, Leaviss J, Al Khalaf M et al. Systematic review, meta-analysis and economic modelling of diagnostic strategies for suspected acute coronary syndrome. Health Technol Assess. 2013; 17. doi: 10.3310/hta17010 10.3310/hta17010 PMC4780933 23331845

[17] Stevenson M, Lloyd-Jones M, Morgan M, Wong R (2012). Non-invasive diagnostic assessment tools for the detection of liver fibrosis in patients with suspected alcohol-related liver disease: a systematic review and economic evaluation. Health Technol Assess.

[18] Cooper KL, Meng Y, Harnan S, Ward SE, Fitzgerald P, Papaioannou D et al. Positron emission tomography (PET) and magnetic resonance imaging (MRI) for the assessment of axillary lymph node metastases in early breast cancer: systematic review and economic evaluation. Health Technol Assess. 2011;15. doi: 10.3310/hta15040 10.3310/hta15040 PMC4781096 21276372

[19] Sutcliffe P, Hummel S, Simpson E , Young T, Rees A, Wilkinson A et al. Use of classical and novel biomarkers as prognostic risk factors for localised prostate cancer: a systematic review. Health Technol Assess. 2009;13. doi: 10.3310/hta13050 10.3310/hta13050 19128541

[20] Pandor A, Eastham J, Beverley C, Chilcott J, Paisley S (2004). Clinical effectiveness and cost-effectiveness of neonatal screening for inborn errors of metabolism using tandem mass spectrometry: a systematic review. Health Technol Assess.

[21] Kaltenthaler E, Bravo Vergel Y, Chilcott J, Thomas S, Blakeborough T, Walters SJ (2004). A systematic review and economic evaluation of magnetic resonance cholangiopancreatography compared with diagnostic endoscopic retrograde cholangiopancreatography. Health Technol Assess.

[22] Schünemann HJ, Moja L (2015). Reviews: Rapid! Rapid! Rapid! …and systematic. Syst Reviews.

[23] Leeflang M, Deeks JJ, Takwoingi Y, Macaskill P (2013). Cochrane diagnostic test accuracy reviews. Syst Reviews.

[24] Song F, Khan KS, Dinnes J, Sutton AJ (2002). Asymmetric funnel plots and publication bias in meta-analyses of diagnostic accuracy. Int J Epidem.

[25] Scherer R, Dickersin K, Langenberg P (1994). Full publication of results initially presented in abstracts: a meta-analysis. JAMA.

